# How much do health care providers value a community-based asthma care program? – a survey to collect their opinions on the utilities of and barriers to its uptake

**DOI:** 10.1186/1472-6963-9-77

**Published:** 2009-05-11

**Authors:** Teresa To, Susan McLimont, Chengning Wang, Lisa Cicutto

**Affiliations:** 1 Child Health Evaluative Sciences, Research Institute, The Hospital for Sick Children, 555 University Ave, Toronto, Ontario, Canada; 2 The Institute for Clinical Evaluative Sciences, G1-06-2075 Bayview Ave, Toronto, Ontario, Canada; 3 The University of Toronto, Toronto, Canada

## Abstract

**Background:**

A comprehensive asthma care program (ACP) based on Canadian Asthma Consensus Guidelines was implemented in 8 primary care sites in Ontario, Canada. A survey was distributed to health care providers' (HCPs) to collect their opinions on the utilities of and barriers to the uptake of the ACP.

**Methods:**

A 39-item self-administered survey was mailed to 184 HCPs and support staff involved in delivering the ACP at the end of implementation. The items were presented in mixed formats with most items requiring responses on a five-point Likert scale. Distributions of responses were analyzed and compared across types of HCPs and sites.

**Results:**

Of the 184 surveys distributed, 108 (59%) were returned, and of that, 83 were completed by HCPs who had clinical contact with the patients. Overall, 95% of the HCPs considered the ACP useful for improving asthma care management. Most HCPs favored using the asthma care map (72%), believed it decreased uncertainties and variations in patient management (91%), and considered it a convenient and reliable source of information (86%). The most commonly reported barrier was time required to complete the asthma care map. Over half of the HCPs reported challenges to using spirometry, while almost 40% identified barriers to using the asthma action plan.

**Conclusion:**

Contrary to the notion that physicians believe that guidelines foster cookbook medicine, our study showed that HCPs believed that the ACP offered an effective and reliable approach for enhancing asthma care and management in primary care.

## Background

Asthma is a chronic disease that affects young and old, rural and urban, immigrants and native resident populations. Many countries have developed and promoted asthma management guidelines to minimize treatment variations and to achieve evidence based best clinical practice. Successful uptake of asthma guidelines into clinical practice settings can be met, but with multiple barriers and challenges [1-5]. First, not all health care practitioners who treat asthma cases are aware of the guidelines and second, those who are aware may not consider the guidelines a helpful tool in making treatment decisions [6,7]. Some reasons contributing to the poor uptake of asthma guidelines is that they are often time consuming and not easy to implement, especially in the primary care setting [6,8,9].

In Ontario, Canada we used a community-based participatory approach to design, implement and evaluate a comprehensive evidence based asthma care program (ACP) and to identify predictors of poor patient outcomes [10]. The diverse interdisciplinary team of health care providers' involved in implementing the program were surveyed in order to identify barriers and challenges to the uptake of the ACP, and to collect their attitudes and perceptions towards its utility in the provision of asthma care.

## Methods

### The asthma care program (ACP)

**Figure 1:**
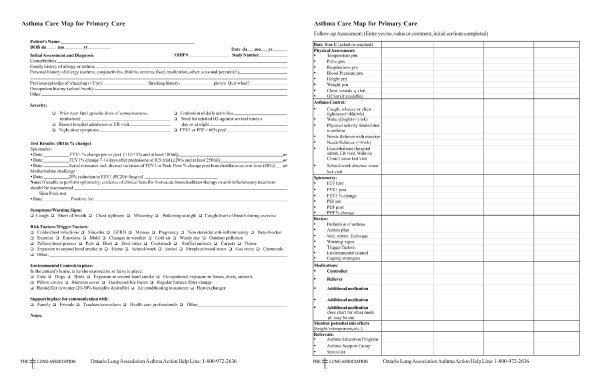
**Asthma care map used in the Primary Care Asthma Pilot Project**.

**Figure 2:**
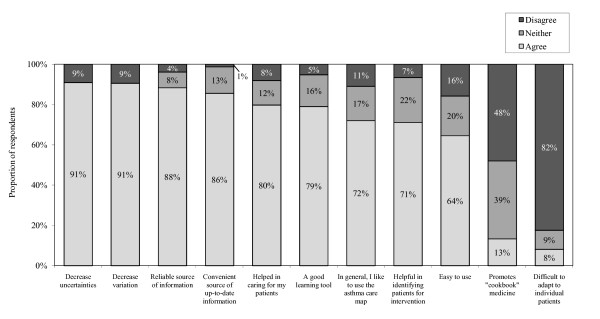
**Benefits to use of the asthma care map (N = 83)**.

The asthma care program (ACP) is based on asthma management standards developed by the Canadian Thoracic Society Canadian Asthma Consensus Guidelines [8,11-14] and consists of 5 components: 1) an asthma care map (Figure 1); 2) a treatment flow chart; 3) program standards 4) a written asthma action plan; and 5) core elements of asthma education. Prior to implementation, the asthma care map was developed by the Ontario Thoracic Society, as part of the Ontario Ministry of Health and Long-Term Care's Asthma Plan of Action, for use by a multi-disciplinary team of primary health care providers (HCPs) as a template for guideline-based management. It incorporates all elements of the Canadian Asthma Consensus Guidelines, including assessment and diagnosis; drug therapy and treatment plan; action plan; and patient education and environmental control. This program is referred to as the Primary Care Asthma Pilot Project (PCAPP) and was conducted between January 2003 and May 2006 in Ontario, Canada. Details of the PCAPP are previously described [10].

### Study setting

The PCAPP was conducted in 8 primary care practices comprising 15 satellite clinics across Ontario, Canada. These sites reside in inner-city, urban and rural communities as well as one isolated Northern Aboriginal community. All of these clinics provide care through a multidisciplinary team of family doctors, nurses, residents, social workers, etc. A designated study coordinator was assigned to each site and was responsible for implementation of the ACP, performing spirometry, providing asthma education to both patients and other health care providers, and coordinating program activities. The study coordinators were Certified Asthma Educators, respiratory therapists or nurses with experience in asthma education.

### Survey study sample

The study population invited to participate in the survey included all 184 HCPs and support staff involved in implementing the ACP at the 15 participating clinics. A 39-item self-administered survey was designed and pilot tested prior to full implementation. Survey participants included family doctors, paediatricians, residents, fellows, nursing staff, respirologists/respiratory therapists, allergists/immunologists, asthma and health educators, and executive directors and clinical/program managers.

### Data collection

Each survey participant involved in the study had previously been assigned a study ID number which was recorded on the survey before distribution. The study coordinator at each of the 15 participating clinics informed HCPs and non-clinicians about the survey during their monthly staff meeting or individually, and distributed the surveys in person or by mail. The surveys were completed and returned to the research team using a self-addressed stamped envelope. Individual respondents at each site were therefore unaware of the responses given by others. In order to increase the response rate, the study coordinators were informed of their individual site response rate and conducted a second distribution of the survey at the following staff meeting in which they emphasized the importance of having the HCPs views of the program. Because of the diversity of staff involved in implementing the ACP some sections were not relevant and therefore not answered by all respondents. For example, administrative staff may not have been involved in providing direct patient asthma care but instead were involved in ensuring patients came in for regular follow-up visits.

### The survey instrument

A search of the literature was done that yielded no direct similarities to the purpose of our survey but did find issues common to asthma intervention in primary care. These issues were used to help guide a focus group consisting of the study coordinators from each site in a small group discussion format to identify components to form the survey. The participants were asked some open ended questions to consider in their small groups (e.g. Looking back since you joined PCAPP what aspects of PCAPP worked well or not well? What are the challenges you experienced and are still experiencing in implementing PCAPP? What components should be changed, what should continue as is, and what should continue but be modified? All things considered what is the utmost important issue to consider in implementing and improving PCAPP?) The moderator then led the discussion to identify survey items, and probed the participants to justify their inclusion/exclusion as well as consider the implications of cost, resources & feasibility in their discussion. The survey was developed based on the recommendations from the focus group to elicit the opinions of HCPs regarding the utility of the ACP and barriers to program uptake. The final 39-item survey was divided into 4 sections (See additional file 1: HCP_Survey.pdf). Section 1 assessed the utility of the asthma care map, the barriers experienced while using it, and preferred formatting. Section 2 assessed the utility of the asthma action plan and flow chart. Section 3 assessed the use of spirometry, peak flow meters (PFM) and medications. Section 4 elicited overall satisfaction with the program. Most items required respondents to indicate their level of agreement on a 5-point Likert scale and other items used a multiple choice format. The survey was first pilot tested at two sites for clarity and then distributed to the remaining sites.

### Analysis

Analysis was conducted using Statistical Analysis Software (SAS) version 9.1. Analysis was conducted on each of the components of the ACP separately as well as on the program as a whole. For each question, percent distributions of the five-point Likert scale (strongly agree, agree, neither agree nor disagree, disagree, strongly disagree) were calculated. Percent distributions are presented in three major groups which combined strongly agree and agree into one category and disagree and strongly disagree into another category, while neither agree nor disagree remained as the third category. The differences in proportions were further analyzed by participating sites and type of health care provider. Given the small number of HCPs at each of the 15 sites, respondents were grouped based on the type of community where the primary care site was located, namely rural, urban or inner-city. Only respondents who had direct clinical involvement with patients with asthma and therefore the ACP were included in the final analyses.

### Ethical approval

The study methodology and materials were reviewed and approved by the Research Ethics Board at the Research Institute, The Hospital for Sick Children, Toronto, Ontario.

## Results

### Demographics

A total of 184 surveys were distributed and 108 (59%) were returned via mail. Only 25 respondents were administrative personnel such as executive directors, clinic managers and secretaries and were excluded from the analyses. Of the 83 respondents who had clinical contact with the patients (i.e. HCPs), the majority (69%) were female and 70% were aged 40 years and above (Table 1). The professions listed by the HCPs included family physicians or residents (52%), nurses or nurse practitioners (37%), and asthma educators or health promoters (11%).

**Table 1:** Demographics of survey respondents

**Characteristic**	**Total Respondents** **N = 83**	Percent*
*Age group*		
		
20–29 years	4	4.9
30–39 years	21	25.6
40–49 years	27	32.9
> 50 years	30	36.6
*(missing = 1)*		

*Female*	53	68.8
		
*(missing = 6)*		

*Provider Type*		
		
Physician/Residents	43	52.4
Nurses/Nurse Practitioners	31	37.8
Asthma Educators	9	11.0

*Practice Type*		
		
Community Health Centre	52	66.7
Group Health Centre	24	30.8
Aboriginal	2	2.6
*(missing = 5)*		

*Practice Location*		
		
Rural	32	41.0
Inner City	30	38.5
Urban	16	20.5
*(missing = 5)*		

* All percentages were adjusted for missing data.

### Evaluation of the asthma care map

**Figure 3:**
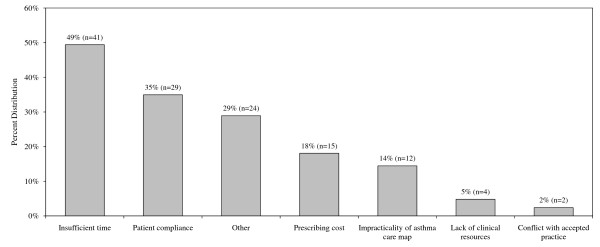
**Barriers to the use of the asthma care map (N = 83)**.

The asthma care map used by HCPs consisted of assessment and diagnosis, severity, objective tests such as spirometry and methacholine challenge test, type of current and prescribed medications, asthma education and environmental trigger management. Figure 2 shows that 72% of HCPs favoured using the asthma care map. The majority of HCPs agreed that it decreased uncertainties (91%) and variation (91%) in management of asthma. The asthma care map used by HCPs during the study period was a one-page double-sided printed document, whereas most HCPs preferred a single page (34%) that could be easily incorporated in the patients' chart as part of the usual care. The majority of the HCPs agreed that the asthma care map was a good learning tool (79%) that served as a reminder and source of up-to-date information (86%). A number of HCPs identified some specific barriers, and 61% of them agreed that it needed some improvement. Figure 3 shows that the most commonly reported barrier was time required to complete it (49%). Other barriers reported were that the asthma care map did not make allowances for managing patients presenting with multiple conditions in addition to asthma, it was not user-friendly, and that there was duplication of the information between the asthma care map and routine documentations on the patient chart. HCPs suggested that it would be useful to incorporate some aspects of the asthma care map into the patient chart as part of the routine clinical documentation in order to minimize time and duplication. There were no statistically significant differences in the responses provided by different types of HCPs or by site location.

### Evaluation of the asthma action plan, flow chart, peak flow meters and medication use

**Figure 4:**
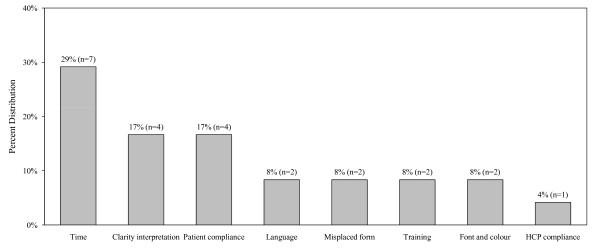
**Challenges to using the asthma action plan (N = 24)**.

**Figure 5:**
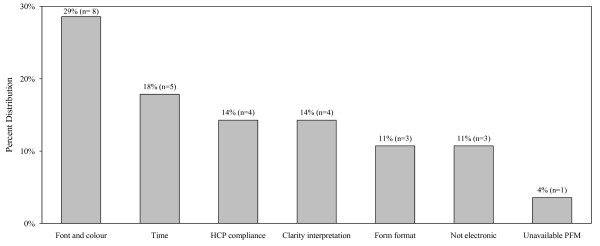
**Challenges to using the asthma treatment flow chart (N = 28)**.

More than one third of the HCPs reported barriers in the use of the asthma action plan (38%) and treatment flow chart (39%) respectively. The specific barriers identified are shown in Figures 4 and 5. Approximately half (51%) of the HCPs reported challenges in using spirometry, which specifically related to training of staff and difficulties in interpretation and application of findings. Teaching peak flow meter use to patients was reported as challenging by 57% of the HCPs, mostly due to lack of patient motivation and time to educate clients. There were no statistically significant differences in responses provided by different types of HCPs or by site location.

### Evaluation of the overall asthma care program (ACP)

In general, the asthma care program (ACP) was well received by most HCPs as they felt that it was appropriate for their patients and practices (96%) and improved the care of their patients (96%). It was also reported by 91% of HCPs that the ACP was implemented with a multi-disciplinary approach. There were no statistically significant differences observed among sites or provider groups. Overall, the most common barriers reported were patient compliance with the regular follow-up visits scheduled at 6- and 12-months and time needed to implement each component of the program.

## Discussion

The Primary Care Asthma Pilot Project (PCAPP) implemented in 15 communities in Ontario showed that participating health care providers (HCPs) in general considered this community-based asthma care program (ACP) valuable in improving care provided to their patients. When developing the program a participatory approach was used to ensure the program was appropriate for a variety of primary care practices and to improve uptake, an approach supported by others [8,15,16]. The current study demonstrated that 96% of HCPs reported that the PCAPP model of care is appropriate for their patients and practices, which supports the importance of using a participatory approach. Work by Christakis *et al *suggest that a change in "behavior" or practice pattern requires the clinicians consider guidelines to be helpful [17]. Our results support this observation in that the majority of the HCPs found the ACP to be useful and would like to see it continue.

Reported in one study, practices characterized by good communication between team members, trust, confidence and physician delegation of asthma management tasks to nurses was associated with higher compliance with asthma guidelines [9]. Overwhelmingly, HCPs (91%) in the current study reported that the ACP was implemented with a multi-disciplinary approach that included the delegation of asthma care tasks to nurses and asthma educators. The participatory developmental phase of the project led to better acceptance of the program by HCPs, which may be directly associated with improvements in asthma health outcomes (such as quality of life and urgent care visits for asthma) and management [10].

Sackett *et al *[18] suggested that clinical or practice guidelines are intended to be "quality-improving strategies" that are user-friendly statements bringing together the best evidence and other knowledge necessary for decision-making about a specific health problem. However, many health care practitioners consider such clinical pathways, guidelines or programs of care as inflexible and not useful in daily clinical practice (often referred to as "*cookbook medicine*") [2,8,17,19,20]. As suggested by Sackett and others, guidelines and care programs must be used as tools in an overall quality improvement plan to meet specific patient population needs [7,18,21]. The challenge, however, is finding the proper balance between clinician autonomy and standardization [22]. Contrary to the common notion that physicians believe that guidelines foster cookbook medicine, only 13% of our participating HCPs were of the opinion that the asthma care map promoted cookbook medicine, the rest believed that it improved care process. Several earlier studies have also reported this observation [23,24].

The literature suggests that implementation of clinical care programs or pathways can increase awareness of best practices and evidence based practice [6,22,25,26]. Although the majority of HCPs agreed that the asthma care map decreased uncertainties (90%) and variation (91%) in the management of asthma, our survey did not include a component to further explore how HCPs felt the PCAPP accomplished this. As our survey was only implemented at the end of the study, we were unable to measure directly the change in knowledge uptake or practice pattern. However, our outcomes assessment in patients enrolled into the program showed that the use of asthma action plans, spirometry and dissemination of asthma education materials increased at both the 6- and 12-month follow-up visits [10]. As well, the acute health care use such as emergency department and hospitalization in our patient population decreased from baseline to 12-month follow-up. These improvements in both short and long-term patient outcomes could have only been achieved by better asthma care provided by HCPs and therefore may serve as indicators of modifications in health care provider practice patterns.

## Conclusion

We conclude that our study suggests that the asthma care program (ACP) offers a well-accepted approach for enhancing asthma care and management in primary care. While in general, health care providers considered the ACP offers a comprehensive approach for enhancing asthma care and management in primary care, they also identified the extra time required to follow through each component as a challenge to a busy primary care setting. Future studies should look into different mechanisms to foster a culture of support to health care providers for adoption of evidence-based practice and health care quality improvement.

## Competing interests

The authors declare that they have no competing interests.

## Authors' contributions

TT conceived of the study, and participated in its design and coordination and helped draft the manuscript. SM participated in the design and coordination of the study and drafted the manuscript. CW performed the statistical analysis and helped to draft the manuscript. LC helped to draft the manuscript. All authors read and approved the final manuscript.

## Pre-publication history

The pre-publication history for this paper can be accessed here:



## Supplementary Material

Additional file 1 **The PCAPP Health Care Provider Survey**. Instrument used to collect data from health care providers. Click here for file
