# Supplementary material for: How much do health care providers value a community-based asthma care program? – a survey to collect their opinions on the utilities of and barriers to its uptake
Source: BMC Health Serv Res. 2009 May 11;9:77. doi: 10.1186/1472-6963-9-77 (PMC2689182; doi:10.1186/1472-6963-9-77)
Supplement: Additional file 1 — The PCAPP Health Care Provider Survey. Instrument used to collect data from health care providers. [file 1472-6963-9-77-S1.pdf]

SickKids

ID: __________

The Primary Care Asthma Pilot Program (PCAPP):
Development, Implementation and Evaluation of Outcomes

Health Care Practitioner Survey

I am inviting you to take part in evaluating the Primary Care Asthma Pilot Program, which was implemented at <Name of Site> during January 2003 to present, as part of the above-referenced research project. The goal of the PCAPP Project is to evaluate whether the use of an evidence-based asthma patient care protocol will lead to improved asthma patient management and outcomes. The program utilizes an asthma care map, action plan and flow chart. Your input or response to this survey will help us determine the utility of the asthma care map as well as what components of the program work, and what components need to be improved.

Please take a few minutes to complete this survey. Your comments and suggestions are critical to the evaluation of the program. Please return your completed survey to the study principal investigator, using the enclosed stamped self addressed envelope. If you have questions or require further information, please contact Dr. Teresa To, the study principal investigator at (416) 555-5555, by e-mail at teresa.to@sickkids.ca or Ms. Jane Doe the project manager at (416) 555-5555. We thank you in advance for your collaboration

Please answer the following questions about yourself:

Your age is within the age group:

- ○ 20-29 years
- ○ 30-39 years
- ○ 40-49 years
- ○ > 50 years

Sex: ○ Male ○ Female

Position:

- ○ Nursing staff
- ○ Resident
- ○ Respiratory Therapist
- ○ Allergist/Immunologist
- ○ Family Doctor
- ○ Asthma Educator
- ○ Executive Director
- ○ Paediatrician
- ○ Respirologist
- ○ Fellow
- ○ Nurse Practitioner
- ○ Health Educator/Promoter
- ○ Clinical/Program Manager
- ○ Other ______________________ please specify

ADMINISTRATIVE STAFF WHO HAVE NOT BEEN INVOLVED IN THE CLINICAL ASPECT OF PCAPP, PLEASE GO TO "SECTION D" AND ANSWER QUESTIONS THAT ARE APPLICABLE

SECTION A (administrative staff → go to section D)

The questions in this section are about the Asthma Care Map (ACM). Please check the most appropriate choice for each statement below reflecting your opinion of the ACM.

	Strongly Agree	Agree	Neither Agree nor Disagree	Disagree	Strongly Disagree
1. *ACM* is a convenient source of up-to-date medical information for managing asthma	○	○	○	○	○
2. *ACM* is a reliable source of information for managing asthma	○	○	○	○	○
3. *ACM* promotes "cookbook" medicine	○	○	○	○	○
4. *ACM* is rigid and therefore is difficult to adapt to individual patients	○	○	○	○	○
5. *ACM* is easy to use	○	○	○	○	○
6. ACM has helped me in caring for my asthma patients	○	○	○	○	○
7. *ACM* is helpful in identifying patients for whom a specific intervention is appropriate	○	○	○	○	○
8. *ACM* is a good learning tool	○	○	○	○	○
9. In general, I like to use *ACM*	○	○	○	○	○

10. In your opinion and experience with using the *ACM*, would you say that its use would:

a) decrease uncertainties in patient management

- ◯ Yes
- ◯ No, please specify ______________________________

b) decrease variation in patient management between practitioners

- ◯ Yes
- ◯ No, please specify ______________________________

11. What barriers have you encountered while using the *ACM*?

- ◯ Lack of clinical resources
- ◯ Insufficient time
- ◯ Conflict with accepted practice
- ◯ Impracticality of care map
- ◯ Prescribing cost
- ◯ Patient compliance
- ◯ Other, please specify ____

12. Which format would you prefer for the ACM?

- ◯ Detailed text
- ◯ Short summary (2-3 page)
- ◯ Flow chart/algorithm
- ◯ Single page – check list
- ◯ Current format
- ◯ Electronic
- ◯ Personal Digital Assistant /Palm PDA
- ◯ Other, please specify ____

13. Please identify whether you found the following sections of the ACM useful, obvious, controversial or confusing (see enclosed ACM for reference)

SECTION	Useful	Obvious	Controversial	Confusing
a) Assessment and Diagnosis	◯	◯	◯	◯
b) Severity	◯	◯	◯	◯
c) Tests	◯	◯	◯	◯
d) Medications	◯	◯	◯	◯
e) Spirometry/Peak flow Meter/ Methacholine challenge	◯	◯	◯	◯
f) Environmental Control	◯	◯	◯	◯

14. **Do you have any suggestions for integrating the ACM in the patient's chart to avoid duplication in documentation?**

____

____

15. **Does the ACM need improvement?**

○ Yes ○ No

→ If Yes, please specify ________________________________

SECTION B (administrative staff → go to section D)

The following are questions about the action plan, and flow chart (see enclosed action plan and flow chart for reference)

16. **Action Plan**

a) Were there barriers to using the Asthma Action Plan?

○ Yes ○ No

→ If Yes, please specify ________________________________

b) Does the action plan need improvement?

○ Yes ○ No

→ If Yes, please specify ________________________________

17. **Flow chart**

a) Were there barriers to using the flow chart?

○ Yes ○ No

→ If Yes, please specify ________________________________

b) Does the flow chart need improvement?

○ Yes ○ No

→ If Yes, please specify ________________________________

SECTION C (administrative staff → go to section D)

The following questions are about Spirometry, Peak Flow Meter and Medications.

18. Spirometry

a) Were there challenges in using Spirometry?

- ○ Yes
- ○ No

→ If Yes, please select <u>or</u> specify below

- ○ Reporting results on care map
- ○ Training of staff
- ○ Appropriate equipment
- ○ Other ____________________

19. Peak Flow Meter (PFM)

a) Were there challenges in educating patients on the use of the Peak Flow Meter?

- ○ Yes
- ○ No

→ If Yes, please select <u>or</u> specify below

- ○ Reporting results on care map
- ○ Availability of PFM
- ○ Availability of mouthpieces
- ○ Patient motivation
- ○ Other ____________________

20. Medications

Do you have any issues regarding the free medications provided by PCAPP?

- ○ Yes
- ○ No
- ○ Inapplicable

→ If Yes, please specify ____________________

SECTION D

The following questions are about different aspects of PCAPP, please check the most appropriate choice for each statement below reflecting your opinion

	Strongly Agree	Agree	Neither Agree nor Disagree	Disagree	Strongly Disagree
21. In general, PCAPP improved asthma care in the clinic	○	○	○	○	○
22. PCAPP model of care is appropriate for my patient population	○	○	○	○	○
23. PCAPP improves my patients' access to asthma care. (e.g. they could call anytime they had SOB or wheeze)	○	○	○	○	○
24. Distance is a challenge for my patients to attend the clinic for asthma care	○	○	○	○	○
25. Having an assigned asthma educator is important to the program	○	○	○	○	○
26. PCAPP was implemented with a multidisciplinary approach	○	○	○	○	○
27. Training of clinical staff in the implementation of study tools was satisfactory	○	○	○	○	○
28. Time is a limiting factor for health care providers to implement PCAPP	○	○	○	○	○
29. The action plan is helpful to my patients	○	○	○	○	○
30. Asthma education provided at the clinic is helpful to my patients	○	○	○	○	○

	Strongly Agree	Agree	Neither Agree nor Disagree	Disagree	Strongly Disagree
31. Environmental assessment for my patients is important	○	○	○	○	○
32. Home based asthma care for my patient population is needed	○	○	○	○	○
33. The written educational materials help my patients understand asthma better	○	○	○	○	○
34. Patients' compliance with the 6 month follow up visits is a challenge	○	○	○	○	○
35. Patients' compliance with the 12 month follow up visits is a challenge	○	○	○	○	○
36. Documentation on the ACM is a challenge	○	○	○	○	○
37. Provision of free asthma medications for patients who cannot afford it, is a positive factor in their asthma control	○	○	○	○	○
38. Provision of free aerochambers for patients who cannot afford it, is a positive factor in their asthma control	○	○	○	○	○
39. I would like this asthma care program to continue	○	○	○	○	○

Please feel free to comment on issues addressed in this survey. Your opinion on how to improve PCAPP would be greatly appreciated.

____

____

____

THANK YOU FOR YOUR TIME
